# SiO_2_–TiO_2_ Nanoparticle Aqueous Foam for Volatile Organic Compounds’ Suppression

**DOI:** 10.3390/toxics12020099

**Published:** 2024-01-23

**Authors:** Jintao Yu, Yuning Xuan

**Affiliations:** Shanghai Institute of Chemical Industry Environmental Engineering Co., Ltd., Guangfu Rd.(W) No.2666, Putuo District, Shanghai 200062, China; yujintao829@163.com

**Keywords:** volatile organic compounds, emerging pollutants, aqueous foam suppression, nanoparticle silica, TiO_2_ photocatalysis

## Abstract

Volatile organic compounds (VOCs) are prevalent soil contaminants. During the ex situ soil remediation process, VOCs may overflow from the soil and cause gas to diffuse into the atmosphere. Moreover, some VOCs, such as trichloromethane, are categorized by the EPA as emerging contaminants, imparting toxicity to organs, and the endocrine and immune systems, and posing a huge threat to human health and the environment. To reduce VOCs’ emissions from contaminated soil, aqueous foam suppression is a prospective method that provides a durable mass transfer barrier for VOCs, and it has been widely used in odor control. Based on an aqueous foam substrate, in order to enhance the foam’s stability and efficiency of suppression, SiO_2_–TiO_2_-modified nanoparticles have been used as stabilizing agents to improve the mechanical strength of liquid film. The nanoparticles are endowed with the ability to photocatalyze after the introduction of titanium dioxide. From SEM imaging, IR, and a series of morphological characterization experiments, the dispersibility of the SiO_2_–TiO_2_-modified nanoparticles was significantly improved under the polar solvent, which, in turn, increased the foam duration. The foam dynamic analysis experiments showed that the foam liquid half-life was increased by 4.08 h, and the volume half-life was increased by 4.44 h after adding the novel synthesized nanoparticles to the bulk foam substrate. From the foam VOC suppression test, foam with modified nanoparticles was more efficient in terms of VOCs’ suppression, in contrast with its nanoparticle-free counterparts, due to the longer retention time. Moreover, in a bench-scale experiment, the SiO_2_–TiO_2_ nanoparticles foam worked against dichloroethane, n–hexane, and toluene for almost 12 h, with a 90% suppression rate, under UV irradiation, which was 2~6 h longer than that of UV-free SiO_2_–TiO_2_ nanoparticles, the KH–570-modified nanosilica foam, and the nanoparticle-free bulk foam. XPS and XRD results indicate that in SiO_2_–TiO_2_ nanoparticles, the proportion of titanium valence was changed, providing more oxygen vacancies compared to raw titanium dioxides.

## 1. Introduction

Ex situ schemes account for a large proportion of soil remediation projects and refer to soil transfers that may disturb the soil’s ambient surroundings. Some contaminated sites are liable to suffer from pollutant leaking if trenching or digging works are carried out. The volatilizing vapors in VOCs pose an intangible threat to human health, which may cause damage to the human respiratory system and blood circulation [1,2,3,4,5]. Therefore, it is essential to reduce the effusion of such organic contaminants. At present, membranes and adsorbents are considered two reliable materials for VOCs control [6,7,8,9,10], both of which are physical barrier agents. Membrane materials include an inflatable membrane structure; these membranes can create a mass transfer barrier against volatile gases on the soil surface [11]. Some adsorbents, such as porous materials, have abundant adsorption sites that provide the ability to contain gas molecules [12]. Although these two materials are efficient in terms of VOCs control, they still lack applications in contaminated soil plots. Moreover, their undegradable characteristics make it difficult to dispose of them after the remediation project is completed.

Stabilizing aqueous foam is currently one of the environmentally friendly materials used for volatile gas control and the suppression of pungent odors [13,14,15,16]. Compared with membrane materials and adsorbents, aqueous foam has inimitable fluidity and flexibility, and strong degradability. The barrier to VOCs that aqueous foams contain is mainly manifested by the ‘circuitous path’ generated inside the foam and the sealing of the liquid carried by bubble film [13,17,18]. As the foam carpet covers contaminated soil, liquid draining occurs simultaneously, making the liquid film thinner and thinner, which undermines the VOC suppression performance [19,20,21,22]. Modified components should be appended to the substrate to improve the stability of foams, which further prolongs the effective suppressing time [23,24,25]. In addition to some common additions to the foam formula, such as lamellae stabilizers, viscosities, or bi-surfactants [20], nanoparticles are likewise regarded as effective ingredients, which can curb the possibility of the foam collapsing by forming a coalescence barrier [26]. Moreover, the expanded specific surface area and plentiful hydroxide radicals on nanoparticles can undergo functional modification. In recent reports, a series of hydrophobic groups have been selected to modify the hydroxyl on the surface of nanosilica [27,28,29,30,31]. The strong emerging dispersion of the post-modified silica can improve multifunctionalities, such as stabilizing emulsion, supercritical extraction, or oil removal. Although the method described above has significantly improved the stability of foam film, the process of foam drainage due to gravity is unavoidable. Since the ability of aqueous foam to blcok VOCs mainly comes from the liquid sealing effect of the liquid film, when the foam gradually drains to form an unstable foam skeleton, its ability to block VOCs is significantly weakened. Therefore, with the foam skeleton framing, the time needed for the foam to block VOCs can be effectively extended if a layer of hydrophobic absorbent materials are attached to the film interface.

The vapor recovery agent, which is coupled with the vapor-suppressing scheme, can enhance the suppressing performance of VOC-contaminated soil [32]. As a novel vapor recovery material, nano titanium dioxide(nano-TiO_2_) possesses the capacity to decompose VOCs because of its photocatalysis performance [33]. In a bench-scale experiment, a SiO_2_ core–TiO_2_ shell composite nanoparticle was shown to enhance the photocatalysis of nano-TiO_2_ [34]. These composite particles are widely used in the environmental or medical fields, have been proven to contain no toxicity, and have no risk of causing secondary pollution [35]. When assembled with the nanoparticle described above, the stabilizing aqueous foam demonstrates a bi-functional property that involves a mass barrier of molecular VOCs, as well as VOC degradation. Moreover, nanoparticles modified by hydrophobic groups show superior dispersibility, meaning they can uniformly distribute on foam lamellae and form an intensive layer to restrain coalescence.

In summary, by adding nanoparticles with functional photocatalytic and improved hydrophobicity to the base of the foam-stabilizing substrate, the particles can spontaneously arrange themselves at the liquid film–air interface, and still retain their original form in a dry foam whose liquid film is completely drained. Ultimately, the particles can create a strong foam skeleton barrier for VOCs’ suppression under intermolecular force.

## 2. Materials and Methods

### 2.1. Samples’ Preparation

#### 2.1.1. Preparation of Bulk Foaming Solution

To prepare a substrate solution, sodium dodecylbenzene sulfonate (SDBS) and N–lauryl sarcosinate sodium (NLSS) were mixed homogeneously at a molar ratio of 7:3 in a total concentration of 0.1 mol/L. After adding 6 vol% triethanolamine, 7 vol% glycerol, 3 g/L Xanthan gum, and 0.3 mol/L ZnSO_4_ into the hybrid substrate solution, ultrasonic mixing was carried on for 15 min to obtain a bulk foaming solution. The bulk foaming solution served as a substrate for the SiO_2_–TiO_2_ nanoparticle foam. All test samples were sourced from Aladdin.

#### 2.1.2. Preparation of Trimethyl Silane (TMCS)-Modified Nanosilica Particles [36] 

Briefly, 25-nanometer silica was added to 15 vol% TMCS n–Hexane or ethanol aqueous solution with a concentration of 50 g/L at ambient temperature. Then, it was activated by sodium hydroxide, and adjusted to ca. pH 9. A further experimental operation was launched after changing the brand-new solution, while the reaction condition was at 60 °C for 2~4 h or more. The post-reaction product was soaked in an n−hexanol solution for 8 h, centrifuged, and dried at 60 °C for 4 h at atmospheric pressure. A gradient heating program was followed to prepare a pure TMCS-modified nanosilica particle with an 80 °C heating program for 3 h and a 100 °C heating program for 2 h. 

#### 2.1.3. Sodium Stearate (SS)-Modified Nanosilica Particle Preparation

Briefly, 10 g of 25-nanometer silica was added in 100 mL deionized water and dispersed adequately in a 250 mL beaker. Sodium stearate with an additive amount of 3.5 g was added to the beaker under 80 °C heating for 40 min in a thermostatic water bath, then cooled to ambient temperature after the reaction was in equilibrium. The after-treatment solution was transferred to a centrifuge tube and centrifuged at a rotation speed of 3000 r/min for 5 min, and then washed with alcohol once and deionized water twice. Hydrophobic modified nanosilica was obtained after drying and centrifugation. 

#### 2.1.4. Preparation of γ–Methacryloyl Oxy Propyl Trimethoxysilane (KH–570)-Modified Nanosilica Particles

Birefly, 10 g nanosilica and KH–570 (15 wt%) were mixed and dispersed adequately in 100 mL deionized water, followed by heating at 80 °C for 12 h in a thermostatic water bath. The final products were washed three times in ethanol, then centrifuged and dried at 100 °C for 2 h. 

#### 2.1.5. Preparation of SiO_2_–TiO_2_ Nanoparticles [34] 

SiO_2_–TiO_2_ nanoparticles were synthesized by adding tetrabutyltitanate dropwise to the hydrophobic nanosilica substrate. The reaction took place in 100 mL ethanol, and 5 g hydrophobic nanosilica was added into the solution. The total dosage of tetrabutyltitanate was 15 vol%. The hydrophobic nanosilica was selected from Section 2.1.2, Section 2.1.3 and Section 2.1.4 by a series of characteristics, as follows. By dropping tetrabutyltitanate constantly, hydrolysis happened on the site of silanol functional group, which is exposed on the hydrophobic nanosilica surface. After an ultrasonic dispersion at ambient temperature for 30 min, the post-reaction product was washed 3 times in deionized water, then centrifuged and dried at 100 °C for 2 h.

#### 2.1.6. Preparation of SiO_2_–TiO_2_ Nanoparticle Foam

Briefly, 0.5 g SiO_2_–TiO_2_ nanoparticles were added into 100 mL of bulk foam solution, prepared as detailed in Section 2.1.1, in a spotless beaker (200 mL), and dispersed by ultrasonic for 15 min. The solution must be foamed immediately when it is dispersed uniformly.

### 2.2. Characteristics

#### 2.2.1. Hydrophobic Performance 

The hydrophobic property of nanosilica after surface modification is strongly related to its lipophilicity, and the degree of lipophilicity, which is related to methanol consumption, is a common index used to evaluate the abundance of hydrophobic functional groups on the surface of nanosilica. A higher lipophilic degree means more methanol consumption, reflecting a strong lipophilicity and outstanding hydrophobic performance [35,36]. According to the relative experimental method, 0.2 g of well-prepared hydrophobic nanosilica particles were added into a beaker containing 50 mL of deionized water. As the methanol was slowly dripped into the beaker under magnetic stirring (200 r/min), the modified nanoparticles floated on the surface of the pure water, and infiltrated gradually as the methanol was added dropwise. The insufficient amount of methanol could only cause part of the nanosilica to infiltrate. When all the particles penetrate totally into the methanol−water phase, the consumption of methanol (V, mL) could be recorded. The calculation method is shown as follows:

(1) $$V_{m}=\frac{V}{50+V}\times 100\%$$

where V is the consumption of methanol; V_m_ is the degree of lipophilicity, which is a dimensionless unit. 

#### 2.2.2. Dispersion Performance 

To evaluate whether the hydrophobic nanosilica particles have outstanding dispersibility, the dispersion performance of the material was assessed. When the particle size distribution is similar, the sedimentation rate of ultrafine powder can faithfully reflect the dispersion of the particles [37], and the sedimentation half-life is also an effective method of reflecting the sedimentation rate [38]. Briefly, 0.2 g of modified nanosilica particles were placed in a vial containing 25 mL of ethanol solution and dispersed ultrasonically for 15 min; they then settled statically on the horizontal plane. An infrared detector was equipped to monitor the sediments’ height in dispersions. The durations were recorded when the sediments’ height settled down to half their original height. 

#### 2.2.3. Stability Performance 

The experiment mainly utilized foam volume half-life (t_1/2_^V^) and foam drainage half-life (t_1/2_^L^) to characterize foam stability. Their specific definitions are as follows.

Half-life of foam volume (t_1/2_^V^): the time it takes for the volume of foam to decay to half of the initial volume of foam. 

Foam drainage half-life (t_1/2_^L^): the time it takes for foam film to drain until the drainage volume reaches half that of the initial foaming solution. 

#### 2.2.4. VOC-Suppressing Performance 

**Figure 1:**
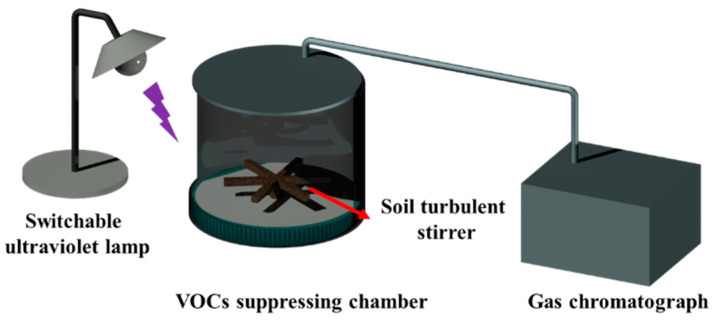
System for evaluating the VOC-suppressing foam.

The suppressing performance was tested using a bench-scale testing system (Figure 1). The testing system contained a switchable UV lamp and a foam VOC-suppressing chamber, and was combined with an Agilent 5977C gas chromatograph–mass spectrometer (GC–MS) which came from the Agilent Technologies Co., Ltd. (Shanghai, China). A soil turbulent stirrer was settled at the bottom of the acrylic jar. Throughout the evaluation process, the stirrer was turned on and mixed the standard soil and contaminants homogenously, with a stirring speed of 50 rpm. After mixing the 1.5 mL volatile organic compounds with 50 g of standard soil in the sealed acrylic jar, the surface was coated with a 4 cm thick aqueous foam, and the foam-covered VOC-contaminated standard soil in the sealed jar was placed into a switchable ultraviolet chamber equipped with a VOC-releasing GC–MS monitor. The chamber was exposed under a 30 W power UV with a wavelength of 254 nm. The gas in the VOC-releasing chamber was extracted every 30 min, and was measured by gas chromatography to obtain corresponding signal values. The relevant software automatically integrated those signals into the peak area (A_i_) as the experimental points were recorded.

The VOCs’ suppression rate at the ith moment is calculated by using the following formula:

(2) $$\theta_{i}=\left(1-\frac{\sum_{1}^{i}A_{i}}{\sum_{1}^{n}A_{i}}\right)\times 100\%$$

where A_i_ represents the response signal integrating the peak area at the ith point. *n* represents the total number of test points. For evaluation of the VOCs’ suppression, this experiment refers to the evaluation criterion of existing studies [14]. We adopt a duration with a suppression rate of 90% (abbreviated to t_90_), and an 80% suppression duration time (t_80_) is similarly used in the suppression assessment.

#### 2.2.5. VOCs Degradation Performance

VOCs contaminant solution with a concentration of 5 mg/L was prepared by diluting the experimental standard agents including dichloroethane, n–hexane, toluene, and trichloromethane. Then, 30 mL of the standard solvents described above were added to a 50 mL vial, and the cap was sealed. Birefly, 0.1 g SiO_2_–TiO_2_ nanoparticles were added into each vial containing the contaminants. The test was carried out under the conditions of UV light or in a UV-free environment, and the concentration was tested by headspace gas chromatography–mass spectrometry eventually. The UV exposure time was 4 h, with a wavelength of 254 nm and a radiation intensity of 70 μw/cm^2^.

#### 2.2.6. Oil Resistivity of Foam

In addition to foam volume and foam stability, the oil resistance of foam also has a significant impact on the ability to suppress VOCs [39,40]. Two main ways of defoaming by oil-phase substances are as follows: (1) A portion of micromolecule oil-phase substances can dissolve in the foaming agent, which reduces the effective concentration of the foaming agent directly. (2) Some oil-phase substances that are insoluble or possess high surface activity may contribute to competitive adsorption with surfactants at the gas−liquid interface. The defoaming ability of the oil-phase material is mainly determined by the entering index (*E*) and spreading index (*S*), and the calculating methods are shown in Formulas (3) and (4). Therein, *γ_AW_* (mN/m) is the surface tension of the foaming solution, *γ_OW_* (mN/m) is the interfacial tension of oil−water mixture, and *γ_AO_* (mN/m) is the surface tension of the oil phase. E indicates the strength of the adsorption capacity of the oil-phase substance at the liquid film, and S indicates the strength of the spreading ability of the oil-phase substance on the liquid film surface. When *E* > 0 and *S* < 0, it is proven that the foam has weak oil resistance to the oil-phase substance. The stronger the defoaming ability of the oil-phase material, the worse the oil resistance of the foam to this material.

(3) $$E=\gamma_{AW}+\gamma_{OW}-\gamma_{AO}$$

(4) $$S=\gamma_{AW}-\gamma_{OW}-\gamma_{AO}$$

## 3. Results and Discussion

### 3.1. Modification of Nanosilica

Silica with abundant silanol exposed on the surface was synthesized using the vapor phase method, which can provide functionalization sites for surface modification. Some silane coupling agents such as KH–570 can easily react with silanol to form a stable hydrophobic tail. The modified silica has a specific hydrophile–lipophile balance (HLB) [41]. When adjusting the quantity ratio of hydrophobic agents and raw silica, the modified silica showed various dispersion characteristics under different solutions. With the hydrophobic groups partly modified on the surface of the silica, dispersion characteristics were significantly improved, promoting the subsequent synthesis of TiO_2_−SiO_2_ nanoparticles.

**Figure 2:**
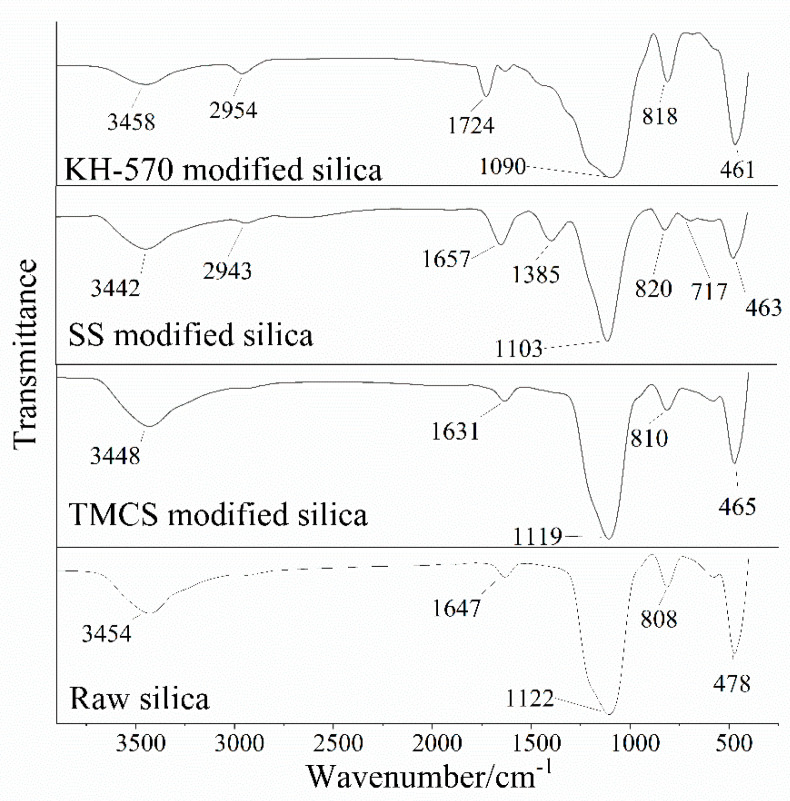
Infrared spectra of nanosilica modified with KH–570, SS, and TMCS.

The modified nanosilica was characterized by Fourier transform infrared spectroscopy with the Thermofisher Nicolet iS5, which is from the Thermo Fisher Scientific (Shanghai, China). The experimental results are shown in Figure 2.

Figure 2 displays the infrared spectra of nanosilica modified with KH–570, SS, and TMCS. Clearly, the characteristic peaks of modified hydrophobic nanosilica were slightly weakened with the shift in the silanol absorption peak (with a wave number of 2800 cm^−1^), except for the TMCS-modified nanoparticles [42]. There are significant shape differences between the modified and unmodified nanosilica at the range of 1750 to 500 cm^−1^. SS has a characteristic peak at 720 cm^−1^, which indicates more than four adjacent –CH_2_– groups [34,43], and a stretching vibration peak at 1300 cm^−1^ belongs to a carbon skeleton of C–C. The more complex circumstance shown in the KH–570 spectrogram has a miscellaneous peak, resulting in a great contrast with the raw silica.

**Figure 3:**
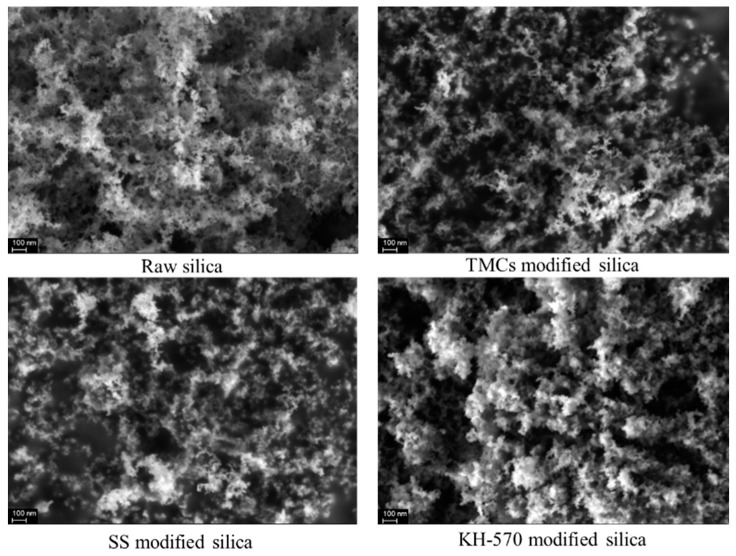
SEM images of hydrophobic nanosilica modified by various groups.

A surface morphology study was carried out with a Zeiss Merlin Compact scanning electron microscope and an Oxford AztecX–Max80 energy-dispersive spectrometer, both are supplied by Beijing Opton Optical Technology Co., Ltd. (Beijing, China), as depicted in Figure 3. The electronic images of raw silica as well as the modified hydrophobic nanosilica were analyzed. Obviously, the raw nanosilica is liable to crosslinking, forming a large and continuous area. The distribution of modified nanosilica is significantly inconsistent. Hydrophobic modified nanosilica with high porosity shows a relatively longer distance between clusters. 

**Figure 4:**
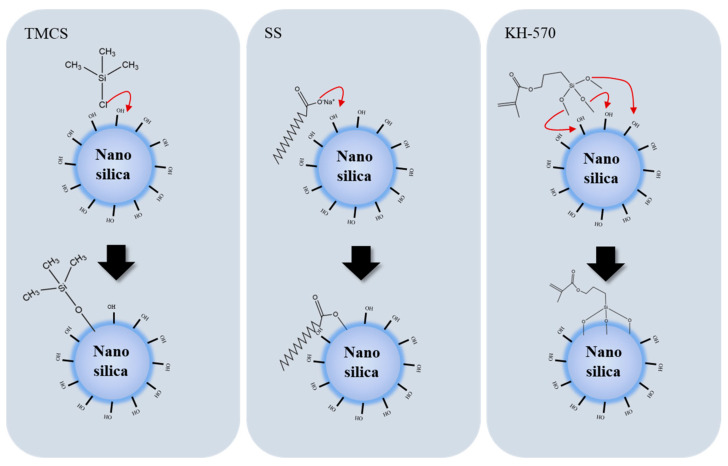
Schematic diagram of the surface modification of hydrophobic nanosilica.

The hydrophobicity of a modified material depends on the hydrophobic properties of the functional groups and the degree of functionalization (Figure 4). TMCS, SS, and KH–570 have different chemical structures, which decide the degree of functionalization efficiency during the surface modification process with nanosilica. TMCS mainly undergoes a substitution reaction between Si–Cl and Silanol, generating volatile HCl. This reaction occurs more readily compared to the forward direction, making it easier to functionalize the surface of nanosilica. The long octadecyl hydrophobic chain of SS leads to significant hydrophobic modification. However, during surface modification, the chain tends to curl, wrapping the COO^−^ at the end of the tail and reducing functionalization efficiency. The Si(OH)_3_ group on the modified end enables a single KH–570 molecule to simultaneously replace three silanol groups on the nanosilica surface with a higher substitution efficiency.

**Figure 5:**
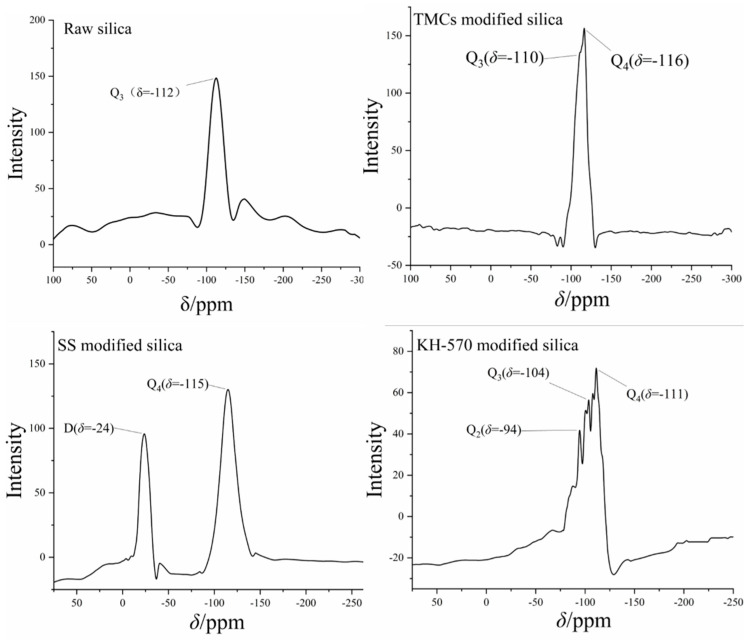
Si–NMR images of hydrophobic nanosilica modified by various groups.

The silicon NMR spectra of three modified nanosilica materials, found by the Oxford MQR, are shown in Figure 5. Chemical shifts in various nanosilica materials are decided by the number of bridging oxygen atoms in each material, which reflect the state of surface substituents [11]. The appearance of Q2 and Q3 peaks indicates that the surface of silica has been modified with other functional groups. According to the spectrum results, KH–570 has a richer variety of peaks and has Q2, Q3, and Q4 peaks simultaneously, causing higher functionalization efficiency. Notably, TMCS shows Q3 and Q4 peaks, suggesting that the silica surface has only a single substitution structure, resulting in lower functionalization efficiency than KH–570. No substitution peak was detected in the SS silicon NMR spectrum, demonstrating that SS undergoes pore adsorption of nanosilica.

Further experiments were carried out to test the characteristic of hydrophobicity alongside the parameter of degree of lipophilicity. Methanol is a typical indicator used to evaluate lipophilicity. By counting different volumes of methanol consumption, the degree of lipophilicity can be calculated using the above formula. According to Table 1, SS has the second-largest degree of lipophilicity at 11.50, while TMCS has the lowest degree of lipophilicity, with a value of 4.94. Thereby, KH–570-modified nanosilica shows the superior hydrophobic performance among the three materials, with a 13.64 degree of lipophilicity, which is consistent with the functionalization efficiency of the Si–NMR reaction.

In addition to maintaining the high specific surface area of nanoparticles, hydrophobic modification of nanosilica can also enhance the dispersion performance of particles in polar solvents. Since the surface of unmodified nanosilica is rich in silanol, the hydrogen bonding effect and the cross-linking effect between nanoparticles are markedly enhanced in polar solvents (such as water or alcohol solvents), affecting the dispersion of particles. Nanosilica with partial surface hydrophobic modification can use the remaining exposed silanol to form hydrogen bonds with solvent molecules, while the hydrophobic groups repel each other; thus, the particles are evenly dispersed in the solvent.

The same circumstance is shown in the sedimentation experiment. As listed in Table 2, KH–570 has the best dispersion performance, with a sedimentation half-life of 13 min, and SS is the second best, with a sedimentation half-life of 10 min. TMCS has the lowest half-life, at 5 min. The half-lives of the three particles are, respectively, 2.5, 5, and 6.5 times that of the raw nanosilica, dramatically prolonging the duration of sedimentation.

### 3.2. SiO_2_–TiO_2_ Nanoparticle Characterization

Based on the above experimental results, KH–570 (15 wt%)-modified nanosilica possesses the best hydrophobic performance, high dispersity in solution, and can prevent aggregation. On the basis of this, a SiO_2_–TiO_2_ composite nanoparticle with a core−shell structure was prepared, and the particle’s characteristics were studied by IR, SEM, and a bench-scale UV degradation experimental test. 

**Figure 6:**
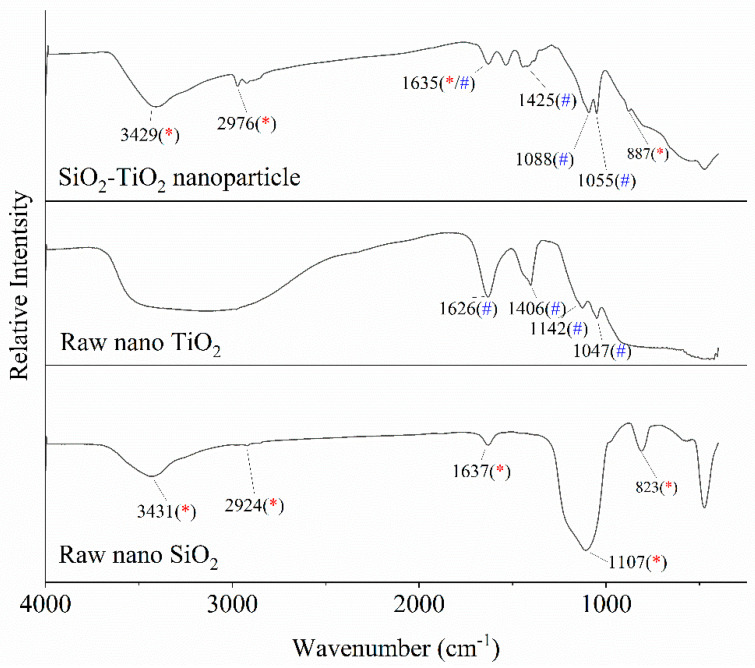
Infrared spectrum of the SiO_2_–TiO_2_ nanoparticle, raw TiO_2_, and SiO_2_ (* marks the characteristic peaks of silica, and # locates the counterparts of TiO_2_).

**Figure 7:**
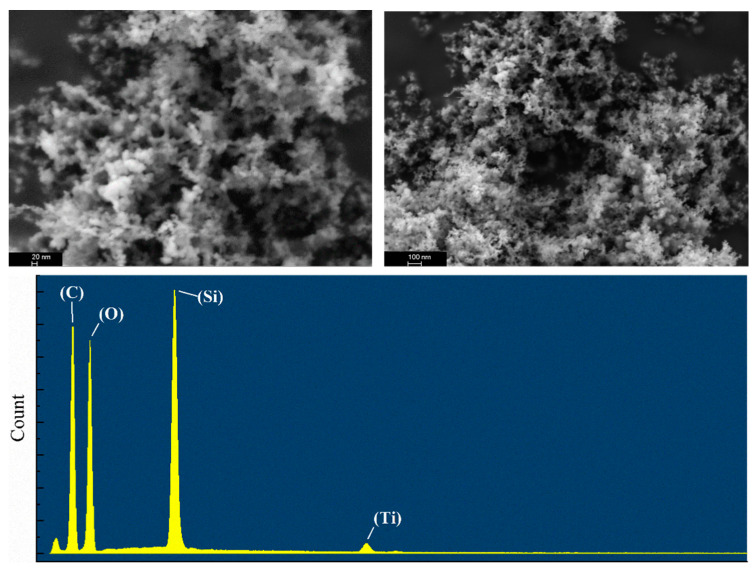

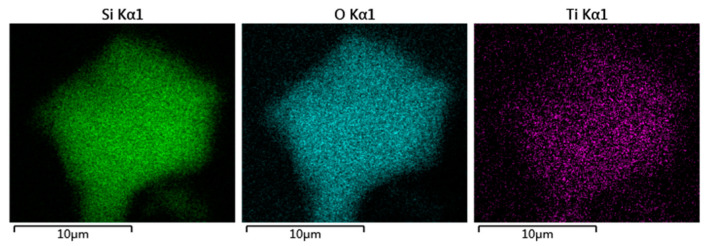
SEM image of the SiO_2_–TiO_2_ nanoparticle.

As the experimental results in Figure 6 show, an overlap of peaks can be observed among the three spectrums in the wavelength range of 3500–2500, 1300–1000, and 850 cm^−1^. It can be preliminarily judged that the TiO_2_−SiO_2_ nanoparticle contains characteristic groups of both SiO_2_ and TiO_2_ components [34], which is consistent with the experimental results of SEM–EDS analysis shown in Figure 7 and Table 3.

In contrast to raw silica, the SEM image in Figure 7 shows that the SiO_2_–TiO_2_ nanoparticle has a porous structure, and the surface layer is covered with a continuously polished phase. According to EDS analysis, the surface layer of the particle was coated with silicon and uniformly trace amounts of titanium.

**Figure 8:**
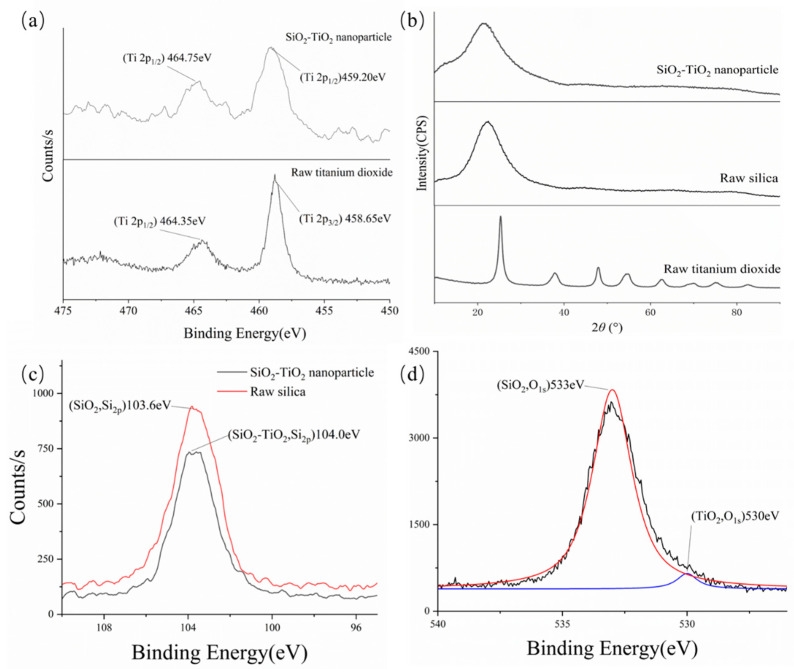
XPS/XRD images of the SiO_2_–TiO_2_ nanoparticle, raw silica, and raw titanium dioxide. (**a**) XPS images of titanium in the SiO_2_–TiO_2_ nanoparticle and raw titanium dioxide; (**b**) XRD images of the SiO_2_–TiO_2_ nanoparticle, raw silica, and raw titanium dioxide; (**c**) XPS images of silicon in the SiO_2_–TiO_2_ nanoparticle and raw silica; (**d**) XPS images of oxide in the SiO_2_–TiO_2_ nanoparticle.

Figure 8b shows that no characteristic peak of TiO_2_ occurred, which indicates that SiO_2_ was still the dominant phase in the SiO_2_–TiO_2_ nanoparticles. From the XPS data in Figure 8a,c,d, titanium and silicon can be simultaneously detected, but the positions of the binding energy peaks were slightly changed. In comparison with raw TiO_2_, the electronic orbit of Ti 2p_3/2_ was shifted by 0.55 eV. The increase in binding energy led to a higher bond dissociation energy in the Ti–O or Ti–Si covalent bonds, making the SiO_2_–TiO_2_ structure more stable; the photocatalytic process could also be carried out under higher-energy UV light instead of visible light, which improved the anti-interference ability of the ambient conditions. The binding energy of O_1s_ showed that most of the oxygen elements form covalent bonds with silicon in SiO_2_–TiO_2_ nanoparticles. Figure 8d displays that the energy of O_1s_ (TiO_2_) in the nanoparticles increased by circa 1.0 eV compared to raw TiO_2_ (529.1 eV) [44], and the enhanced Ti–O bonds may create more oxygen vacancies for electron transfer, thus improving photocatalytic efficiency [45]. In summary, compared to raw TiO_2_, SiO_2_–TiO_2_ needs UV light instead of visible light or simulated solar light. This change causes the materials to undergo photocatalysis under an external trigger, rather than unstable conditions. This characteristic also means that catalysis of foam constituents under solar irradiation can be avoided. Moreover, SiO_2_–TiO_2_ frameworks can provide more abundant active sites on the surface of silica for VOCs’ degradation, because of the stronger binding energy of Ti–O [46,47].

### 3.3. Aqueous Foam Stability

**Figure 9:**
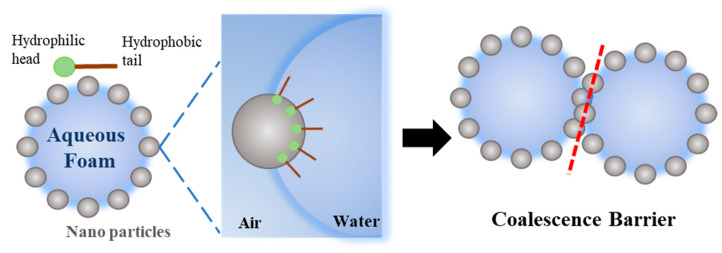
Schematic diagram of the coalescence barrier action of nanoparticles.

When modified nanoparticles are introduced into the stable foam stock solution, the particles will spontaneously move to the air-liquid interface, and the semisphere of particles will be exposed under the air phase due to the hydrophobic groups on the surface of the particles. The exposure area is related to the degree of hydrophobicity. This kind of self-assembled nanoparticle on the surface of the liquid film can form an agglomeration barrier to further prevent the liquid film between foams from coalescing (Figure 9). As a result, the foam continues to maintain a fine size and restrain the Ostwald ripening process.

**Figure 10:**
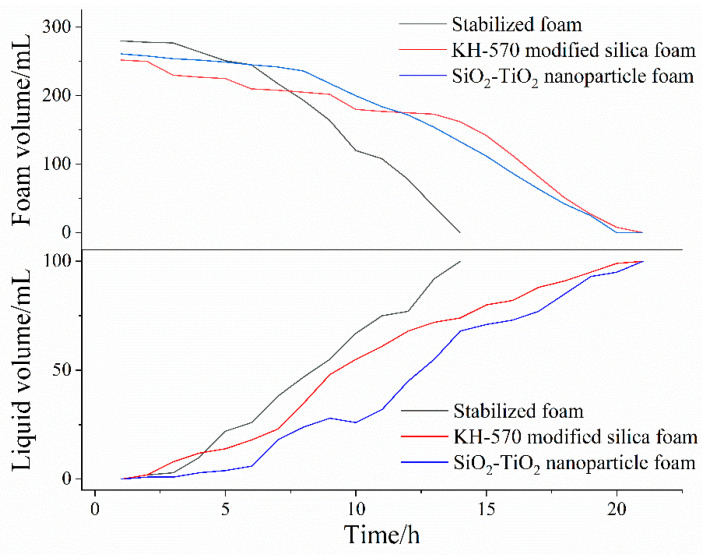
Dynamic foam analysis curve of foam/liquid volume as a function of time.

**Figure 11:**
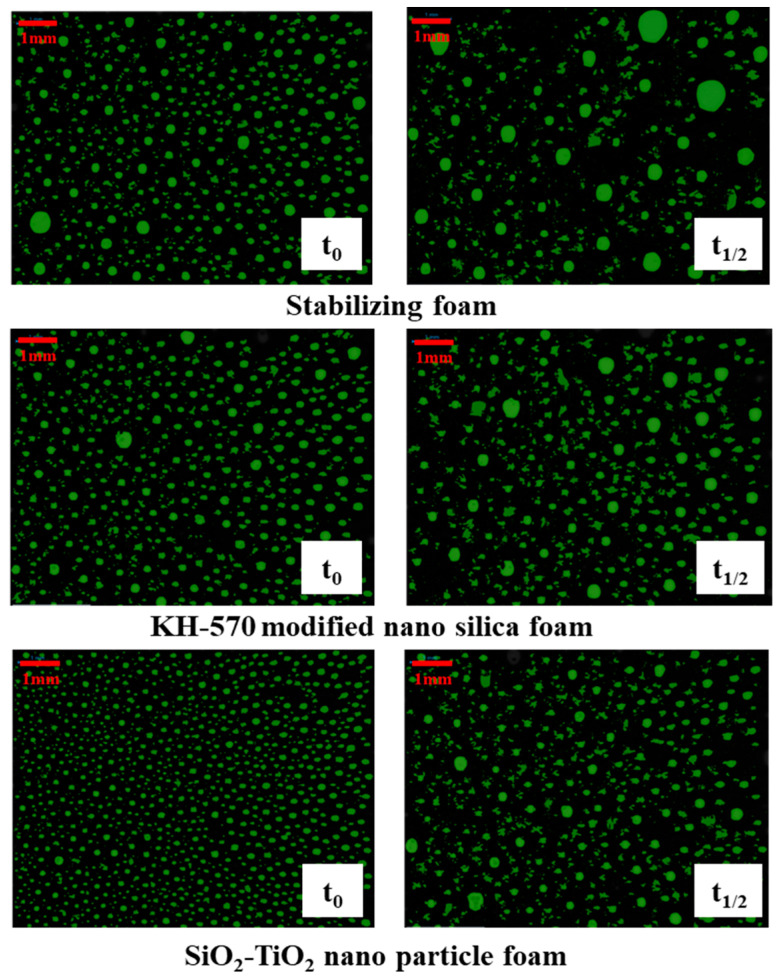
Images of dynamic foam analysis.

A Krüss DFA100–FSM dynamic foam analyzer provided by Krüss Scientific Instruments Co., Ltd. (Shanghai, China) was used to observe the morphology of aqueous foam as a function of time. In the results of foam dynamic analysis test, there were great morphological differences between unmodified and hydrophobically modified particle foams. As shown in Figure 10, the liquid volume half-life and foam volume half-life of each column were measured, as shown in Table 4. Looking at the experimental results, after the addition of modified nanosilica particles, the liquid half-life and foam half-life were significantly improved, indicating enhanced stability. Foam structures with different stages were obtained from the dynamic analysis images in Figure 11. When the SiO_2_–TiO_2_ nanoparticles were added into the stable bulk foam solution, the average size of the bubbles at the foam volume half-life obviously reduced, thus effectively preventing the foam aggregation process, as well as enhancing the foam’s duration. This is consistent with the mechanism of the coalescence barrier.

### 3.4. Aqueous Foam Suppressing VOCs

Aqueous foam can significantly suppress most VOCs by forming a circuitous route for the harmful vapor. The lamellae between the adjacent foam is capable of providing a mass transfer barrier to retard the vapor transition through the vapor solution–volatilization process on the foam membrane surface. However, the suppression ability is completely dependent on the lamellae thickness, which is directly decided by the amount of liquid that the foam membrane can carry. In addition, vapor leakage happens when the adjacent foam coalesces or rearranges, promoting the gas transfer rate and the formation of vacant sites. By adding photocatalytic nanoparticles to the foam solution, the foam membrane was mechanically strengthened and endowed with photocatalytic function [34,48]. Bi-functional foam can make up for the lack of barrier capacity in single liquid film. 

Experiments on foam’s suppressing performance were carried out using the assessment index of suppressing rate, calculated with Formula (2). Each stabilizing foam solution contains 0.5 wt% nanoparticles or nanosilica. Three stems of aqueous foam (unmodified nanosilica foam, SiO_2_–TiO_2_ nanoparticle foam in a UV-free environment, and SiO_2_–TiO_2_ nanoparticle foam under UV light) were used to suppress the following typical VOCs: a chlorohydrocarbon (dichloroethane), a petroleum hydrocarbon (n–hexane), a benzene derivative (toluene), and an emerging contaminant (trichloromethane). All the tests of VOCs were carried out by diluting standard laboratory agents.

In order to reflect the photocatalytic effect of the nanoparticles, a bidirectional experiment, including the conservation of the VOC suppressing ratio as a function of time, was carried out with modified nanoparticles and unmodified nanoparticles. The experimental results are as follows. From the dynamic curve, the t_80_/t_90_ index is presented in Table 4. Compared with unmodified nanosilica, the t_80_ and t_90_ VOC suppressing times of the modified nanoparticle foam were longer than those of the nanoparticles-free stabilizing foam. The SiO_2_–TiO_2_ nanoparticle foam under the irradiation of UV light showed an excellent VOC-suppressing ability on four contaminants. Table 5 shows that the suppression times of the four pollutants are obviously different, depending on the degree of oil resistance of foam materials to different pollutants. From Table 6, all the E indexes of foam solution are greater than 0 in the presence of n–hexane, dichloroethane, and toluene, and S indexes are less than 0, indicating that the four pollutants have a defoaming effect. From the indexes of toluene, we observed that this contaminant is more easily adsorbed on the surface of the liquid film, and forms a refractory liquid droplet, which is consistent with the short duration of toluene’s blocking rate. The experimental data of n–hexane indicate that n–hexane is more easily adsorbed on the surface of the liquid membrane, affecting the effectiveness of the surface active agent. Therefore, the resistance of the foam to n–hexane is weaker than that of dichloroethane, toluene, and trichloromethane.

**Figure 12:**
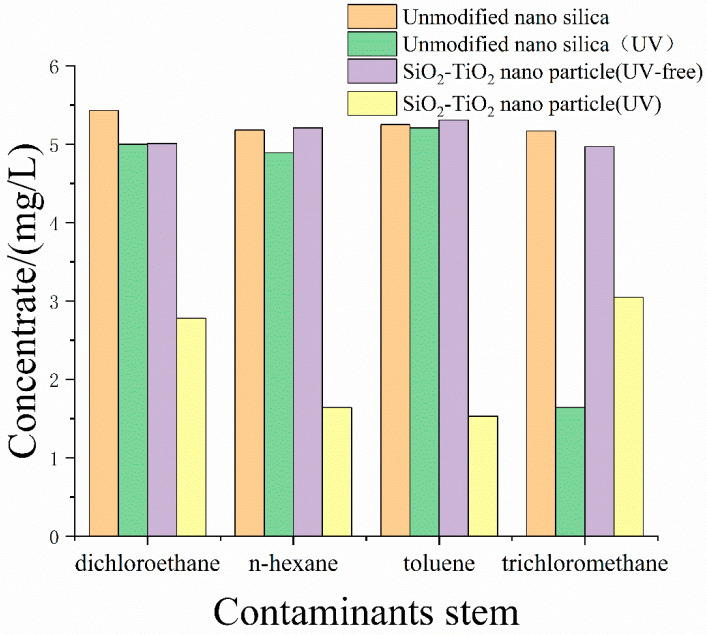
Degradation experiment results of unmodified nanosilica, UV-free nanoparticles, and nanoparticles under UV.

**Figure 13:**
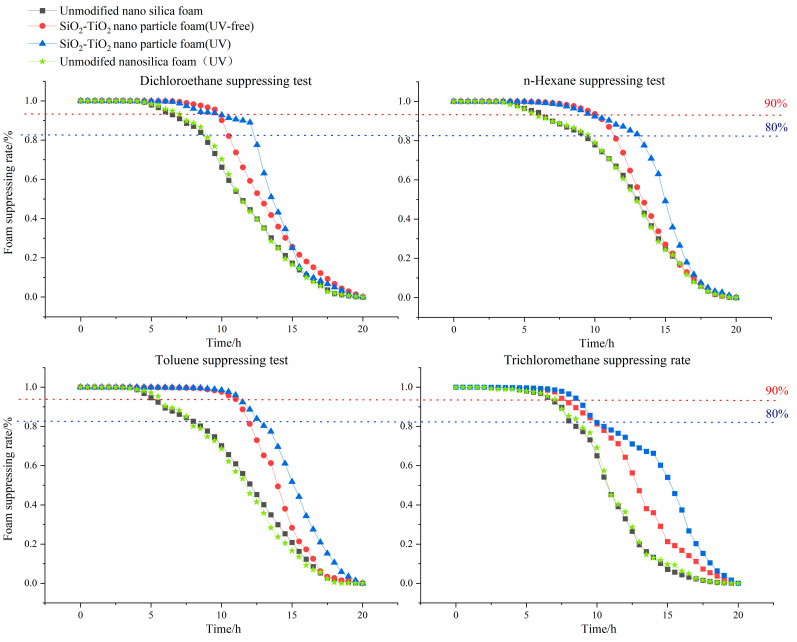
Dynamic suppression rates of aqueous foam with four typical contaminants as.

Convergence barriers were formed during the transfer of nanoparticles to the air-liquid interface of bubble film, which obstructed the gas penetration between foam units. The barriers are also able to strengthen the foam structure, enabling more flexibility to confront outer vapor pressures such as wind. The prolonged retention time increases the suppression time accordingly. At the same time, the titanium in the SiO_2_–TiO_2_ composite nanoparticles plays a role in photocatalysis, causing the amounts of VOCs to be further effectively suppressed. The photocatalytic property of the nanoparticles was characterized by the degradation test. Experiments were performed by headspace gas chromatography–mass spectrometry, and results are shown in Figure 12. Apart from the first three typical VOCs, trichloromethane is easily affected by photochemical reactions and can be degraded without the addition of catalysts, producing hydrogen chloride and phosgene [49]. The introduction of nanoparticles may weaken the degradation caused by the photochemical reaction due to the decrease in light transmittance. Furthermore, as the foam lamellae become thinner because of the film liquid drainage, the foam skeleton keeps its initial frame structure, which gradually emerges from top to bottom; it has then lost the function of mass transfer across the barrier. By adding the photocatalytic nanoparticles in the foam solution, the post-forming skeleton of aqueous foam will be endowed with a function of VOCs’ adsorption-degradation, meaning the dry foam is still able to suppress the VOCs. This theory can be testified by the experimental solution shown in Figure 13, in which the subsequent suppression time t_80_ of UV-irradiated foam is slightly delayed by about 1 h compared to the UV-free case.

## 4. Conclusions

(1) Three silicon surface modifiers: trimethylchlorosilane, sodium stearate, and KH–570 were used to carry out hydrophobic modification of nanosilica. The dispersion of nanosilica after surface hydrophobic modification was significantly improved, and KH–570-modified nanosilica nanoparticles showed superior hydrophobic and dispersion performance, with a lipophilicity degree of 13.64 and a sedimentation half-life of 13 min in ethanol solvents. From the SEM image, the distance between clusters in the nanosilica aggregate was stretched, meaning a porous structure was formed, and there was a more uniform distribution of clusters. Through a lipophilicity test, a sedimentation half-life test and surface morphology characterization, it can be found that the cross-linking between nano-silica modified by a coupling agent is significantly weakened, and the dispersion in polar solvents is stronger, making it suitable as a synthetic substrate of SiO_2_–TiO_2_ nanoparticles.
(2) After the addition of SiO_2_–TiO_2_ nanoparticles, the gas permeability between the foams was significantly reduced due to the aggregation barrier, the smaller initial size of the foam, and the delicate and uniform foam. The liquid volume half-life was increased by circa. 2 h, and the foam volume half-life was increased from circa. 9 to 14 h, which indicates greatly improved stability. The liquid volume half-life and the foam volume half-life of the SiO_2_–TiO_2_ nanoparticle foam are similar those of the KH–570-modified nanosilica, with a 12.54 h liquid volume half-life and a 14.06 h foam volume half-life.
(3) The effective barrier time was assessed to compare the SiO_2_–TiO_2_ nanoparticle foam’s VOC suppression ability under different conditions. The VOC suppression properties of the SiO_2_–TiO_2_ nanoparticle foam (UV), SiO_2_–TiO_2_ nanoparticle foam (UV-free), original nano−SiO_2_ (UV-free), and original nano−SiO_2_ (UV) were decreased in order. Compared with unmodified nanosilica foam, the SiO_2_–TiO_2_ nanoparticle foam showed stronger suppression performances, especially under UV light irradiation, and the indices of suppression (t_80_, t_90_) of dichloroethane, n–hexane, toluene, and trichloromethane were higher than those of the former foam for 2~6 h.

## Figures and Tables

**Table 1:** Lipophilic degree of modified hydrophobic nanosilica.

Materials	Methanol Consumption Volume (mL)	Degree of Lipophilicity
Raw silica	0.6	0.01
TMCS-modified nanosilica	2.6	4.94
SS-modified nanosilica	6.5	11.50
KH–570-modified nanosilica	7.9	13.64

**Table 2:** Sedimentation experiment results of modified hydrophobic nanosilica.

Materials	Sedimentation Half–Life (min)
Raw nanosilica	2
TMCS-modified nanosilica	5
SS-modified nanosilica	10
KH–570-modified nanosilica	13

**Table 3:** EDS element analysis of the SiO_2_–TiO_2_ nanoparticle.

Chemical Element	wt%
O	49.19
Si	9.73
Ti	0.93

**Table 4:** Foam/liquid half-life of various aqueous foams.

Foam Stem	Liquid Volume Half-Life/h	Foam Volume Half-Life/h
Stabilizing foam	8.46	9.62
KH–570-modified silica foam	10.34	14.50
SiO_2_–TiO_2_ nanoparticle foam	12.54	14.06

**Table 5:** VOC suppression evaluation of various nanoparticle aqueous foams.

Foam Type	Stem of Contaminants							
	Dichloroethane		n–Hexane		Toluene		Trichloromethane	
	t_80_	t_90_	t_80_	t_90_	t_80_	t_90_	t_80_	t_90_
Unmodified nanosilica foam	8.89	7.24	9.64	7.08	8.57	5.90	8.57	7.52
Unmodified nanosilica foam (UV)	9.01	7.50	9.55	6.89	8.15	6.42	9.02	7.60
SiO_2_–TiO_2_ nanoparticle foam (UV-free)	10.65	10.05	11.59	10.71	12.11	11.35	10.28	8.47
SiO_2_–TiO_2_ nanoparticle foam (UV)	12.45	11.94	13.32	11.19	13.15	11.84	10.71	9.16

**Table 6:** Entering index and spreading index of various oil−surfactant phases.

Phase	Surface Tension/(mN·m^−2^)	Entering Index (E)	Spreading Index (S)
n–Hexane	23.8	20.9	−12.3
n–Hexane/surfactant	16.6		
Dichloroethane	32.2	2.2	−10.4
Dichloroethane/surfactant	6.3		
Toluene	28.5	14.6	−15.4
Toluene/surfactant	15.0		
Trichloromethane	28.9	14.7	−16.3
Trichloromethane/surfactant	15.5		
Surfactant solution	28.10	−	−

## Data Availability

Our data are unavailable due to privacy.
